# *Acmella oleracea* induced nanostructured Ca_2_Fe_2_O_5_ for evaluation of photo catalytic degradation of cardiovascular drugs and bio toxicity

**DOI:** 10.1016/j.heliyon.2023.e15933

**Published:** 2023-04-29

**Authors:** Neelam Patil Radhika, Malini S, Kalyan Raj, K.S. Anantharaju, Shylaja K. R, Abhishek Appaji

**Affiliations:** a Department of Chemistry, K.S. Institute of Technology, Bengaluru, India; b Department of Chemistry, B.M.S. College of Engineering, Bengaluru, India; c Department of Chemistry, Dayananda Sagar College of Engineering, Bengaluru, India; d Department of Medical Electronics Engineering, B.M.S. College of Engineering, Bengaluru, India; e University Eye Clinic Maastricht, Maastricht University, Maastricht, the Netherlands

**Keywords:** Calcium ferrate, Photocatalytic, Cardiovascular drugs, Antibiofilm, Endodontics, Cytotoxicity

## Abstract

Biosynthesis of nanoparticles is increasingly becoming popular due to the demand for sustainable technologies worldwide. In the present investigation, *Acmella oleracea* plant extract fuelled combustion technique followed by calcination at 600 °C was adopted to prepare nanocrystalline Ca_2_Fe_2_O_5_. The prepared nano compound was characterised using X-ray powder diffraction (XRD), scanning electron microscopy (SEM), Ultra Violet (UV) spectroscopy, Infrared (IR) spectroscopy and its role was assessed for photocatalytic pollutant degradation along with bactericidal action in the concentration range of 1 μg/mL to 320 μg/mL. The photocatalytic degradation efficiency of pollutant drugs Clopidogrel Bisulphate and Asprin used for cardiovascular disorders is around 80% with 10 mg/L photocatalyst. The results showed that the photocatalytic activity increased with rising pH from 4, to 10, along with a significant antibacterial action against *Enterococcus faecalis* bacteria and a slight cytotoxic effect at high concentrations. The antibacterial property was reinforced by Minimum inhibitory concentrations (MIC) and Minimum bactericidal concentrations (MBC) studies with an average value of 0.103 at 600 nm which was further proved by significant *anti*-biofilm activeness. Adhesion tests in conjunction with cryogenic-scanning electron microscopy displayed a morphological change through agglomeration that caused an expansion in nano particles from 181 nm to 223.6 nm due to internalization followed by inactivation of bacteria. In addition, the non-toxicity of nano Ca_2_Fe_2_O_5_ was confirmed by subtle cytological changes in microscopic images of Allium Cepa root cells in the concentration range 0.01–100 μg/mL and a slight inhibition in HeLa cell proliferation indicated by IC_50_ value of 170.94 μg/mL. In total, the current investigation for the first time reveals the application of bio based synthesis of Nano Ca_2_Fe_2_O_5_ to new possibilities in bioremediation namely degrading cardiovascular pharmaceutical pollutants, endodontic antibacterial action and cytological activity.

## 1 Introduction

Extensive growth of medical technology has raised the production and consumption of pharmaceutical molecules. Unscientific disposal or excretion after the intended use, permits them to enter into water resources and emerges as a relevant class of complex contaminant. A persistent chemical stability of these molecules in traces pose a serious threat leading to social concern as they get away with most water treatment plants. The toxicity of these compounds is now well established [1] and therefore the Environmental protection agency (EPA) has issued directives to implement regulations towards sustainable pharmaceutical waste management [2].

Despite many eco-friendly attempts such as highly adsorbing hydrochar [3], microbial peroxide producing cell [4] incorporating microbial electrode, simple bacterial cultures [5] and oxidation [6] that transforms the toxic drugs, Nano material aided photocatalytic degradation of pollution causing drugs is gaining high popularity due to its low cost, ease and efficiency. In addition, zeolitic imidazolate framework embedded with Cobalt based material [7] and ZnO@N-doped Carbon [8] along with Cellulose-zeolitic imidazolate framework [9] has served as an excellent platform for photocatalytic degradation of organic pollutants. Recently, coupling of two metallic nanoparticles such as Co–Ta, Cu–Ti and Cu-diamond nanoparticles has proved beneficial in hemodynamics [10], electroosmotic flow [11] and entropy optimization [12] respectively.

Several nano photocatalytic strategies are designed so far to degrade popular pharmaceuticals Diclofenac [13], Asprin [14], Ornidazole [15], sulfamethoxazole [16] Tetracycline hydrochloride [17] and many more have played a major role in saving many ecosystems worldwide. In view of increasing number of incidences of cardiovascular diseases leading to large consumption of the cardiovascular drugs the focus is now shifted towards photocatalytic degradation of cardiovascular drugs such as salbutamol [18], metoprolol [19] carbamazepine and atenolol [20].

It is noticed that researchers have utilised europium doped titanium dioxide [21] to degrade Clopidogrel bisulphate with P_25_TiO_2_ [22], polymeric film of TiO_2_ [23] and Nano crystalline Pt embedded TiO_2_ nano-tube array [24] to degrade Asprin.

Clopidogrel is used extensively either alone or with Aspirin which metabolises in the liver by cytochrome P450 (CYP) system. They are released to the environment after the intended use and is persistent owing to their extreme structural stability. With this background, a photocatalytic degradation methodology to combat the accumulation of these toxic compounds in natural resources becomes highly essential.

Among the Nano catalysts becoming popular, mixed metal oxides, especially the oxides of Ca with Fe are widely researched in the area of heterogeneous catalysis owing to their large surface area [25], magnetic recoverability [26], electrochemical activity [27] and phase versatility [28].

In recent years, numerous advancements are observed using Calcium Ferrate composites for environmental remediation [29] through photocatalytic degradation as both Calcium and Iron are non-toxic elements, abundantly available and possess a small particulate size arising from strain across the crystalline phases. Its capacity to form excellent heterojunction has given way to exploring statistical modelling of self-assembled CaFe_2_O_4_**/**ZrO_2_ [30] applied to removal of Tetracycline antibiotic from aqua matrix that has inspired many more similar studies which is often driven by generation of reactive oxygen species [31].

In addition, researchers have paired photocatalytic degradation studies with antibacterial applications more often due to the reactive oxygen species generated by these nano catalysts that tend to deplete antibiotic resistance and induce antimicrobial lethality [32]. Hence, this has led to many developments such as tri-phasic nanocomposite [33] and CaFe_2_O_4_ doped CdO heterojunction Nano hybrid [34] materials becoming instrumental in battling microbial resistance and bacterial poisoning where the traditional methods fail.

However, with new pharmaceutical pollutants entering the ecosystem, and large number of microbes developing resistance there is a severe need for novel nanocatalysts with suitable modifications to combat the problem of pharmaceutical pollution as well as microbial infections. In view of this, the current investigation reports the synthesis of Nano Ca_2_Fe_2_O_5_ followed by characterization using XRD, SEM, EDAX, FTIR, UV for determining the average crystallite size, surface morphology, elemental composition, bonds in functional groups and band gap respectively.

The synthesis follows a combustion process fuelled by the extract of *Acmella oleracea,* a flowering herb largely found in central India and is traditionally used for relieving tooth ache.

The prepared compound is utilised to establish the photocatalytic degradation efficiency of Clopidogrel bisulphate and Asprin two commonly used cardiovascular drugs administered together, at varying pH that serve as model pharmaceutical pollutants. In addition, the compound is also used against *Enterococcus faecalis a* bacteria notorious for forming endodonic biofilms with antibiotic resistance which is a public health concern.

In our observation, this is the first report where Nano Ca_2_Fe_2_O_5_ is synthesized using *Acmella oleracea* plant extract by combustion method, used to degrade the cardiovascular drugs. The nano dimenstion of the prepared Ca_2_Fe_2_O_5_ makes it suitable for investigating microorganisms and hence the study combines the validation of antibacterial properties using *Enterococcus faecalis* through planktonic and biofilm studies**.** Biosafety of the prepared Ca_2_Fe_2_O_5_ nanoparticles is ensured by toxicity studies on Allium Cepa root cells and HeLa cell line.

This work will extend a deeper insight and new possibilities into drug waste-water treatment, endodontic antibacterial activity and toxicity analysis.

## 2 Experimental

### 2.1 Reagents

Analytical grade Ca (NO_3_)_2_·4H_2_O, Fe(NO_3_)_3_·9H_2_O and other reagents of high purity was purchased from Sigma Aldrich and hence was used without any additional purification. *Acmella oleracea* plant was bought from certified medicinal plant retailers in Bangalore.

### 2.2 Characterization

The crystal structure of the obtained pure Ca_2_Fe_2_O_5_ was explored using powder X-ray diffraction XRD using Bruker D_8_ Advance Diffractometer equipped with Cu-Kα radiation source fit with a graphite monochromator of wavelength 1.54 Å, in the scanning range of 20°–80° at 3°/min scanning speed to obtain 2θ with a step width of 0.05ᵒ and 60 s duration for each step.

Surface morphology was investigated using Hitachi S-3400 Scanning electron microscopy with an EDAX attachment for elemental analysis. The Fourier Transform-Infrared (FT-IR) spectra is obtained using NEXUS 470, Nicolet, USA in the range of 550 cm^−1^ to 4000 cm^−1^.

UV spectrophotometer (Shimadzu, Japan) was used for all absorbance detection towards band gap calculations and photo degradation analysis.

Fluorescence measurements were carried on using Ultra Perkin- Elmer India, Model - 50,107, LS55(230 V), equipped with a rectangular cell semi micro cuvette of 10 mm having magnetic stirrer and Water circulator.

Computer software Origin Pro 7.5 along with Microsoft Excel was used for plotting and obtaining numerical solutions for the data analysis.

### 2.3 Preparation of ethanol extract of Acmella oleracea plant

The *Acmella oleracea* plant was cleaned in distilled water, dried and crushed using a pestle and mortar. 250 g of coarse dry powder was loaded in the soxhlet apparatus containing ethanol and extraction was carried out for 18 h. The crude ethanol extract was concentrated using rotary evaporator and preserved at 4 °C for future utilization.

### 2.4 Synthesis of medicinal plant mediated Ca_2_Fe_2_O_5_ nano powder

Stoichiometric ratios of primary material 5.687 g of Ca(NO_3_)_2_·4H_2_O and 9.167 g of Fe(NO_3_)_3_·9H_2_O were combined with *Acmella oleracea* plant extract as a fuel in a silica pot and mixed with minimum quantity of distilled water to obtain a homogeneous blend.

**Fig. 1:**
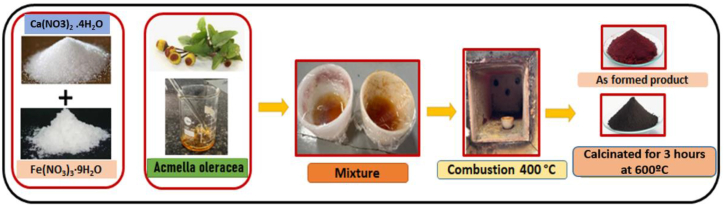
Flowchart representing the synthesis of Ca_2_Fe_2_O_5_ Nano powder.

It was subjected to combustion at 400 °C which led to a quick burst of flames within 2 min. The product was further set for calcination at 600 °C for 3 h. The procedure was repeated with varying volumes of plant extracts in the range of 2 mL–8 mL. Fig. 1 displays a pictorial representation of obtaining the nano product which was characterised using various techniques.

### 2.5 Photo degradation experiments

Photo degradation studies were carried out using pharmaceutical pollutants Clopidogrel and Asprin. As the catalyst showed a mild activity at low concentrations and very little tendency to settle at the bottom during centrifugation at high concentrations, loading of catalyst to each of the drugs was achieved at an optimum concentration.

To 100 mL of each of the drug solution with a concentration of 10 mg/L prepared 20 mL of Ca_2_Fe_2_O_5_ catalyst with concentration of 20 mg/L was added with constant magnetic stirring to reach the adsorption equilibrium. The set up was placed in the dark for 30 min to attain dark adsorption equilibrium and no appreciable degradation was observed when kept in the dark. The photo degradation was triggered through illumination by placing the mixture under a 300 W halogen lamp for 50 min [35]. 5 mL of the drug catalyst suspension was pipetted out at every 5-min interval, centrifuged and the supernatant was immediately subjected to UV–Visible absorption spectroscopy studies to monitor the residual drug.

### 2.6 Mass fragmentation studies

The GC–MS (17A Shimadzu Gas Chromatography) enabled with electron input ionizer technique with a QP-5050 was used to record the mass spectroscopy data. 500 μL/ml mixture obtained at the end of 50 min of degradation was directly injected at a flow rate of 10 μl/min and the parameters were set to get best possible fragmentation with optimum sensitivity.

### 2.7 Fluorescence measurements

Fluorescence emission of Clopidogrel bisulphate and Asprin with concentration 10 mg/L was obtained in the presence of Ca_2_Fe_2_O_5_ catalyst 20 mg/L dispersion in distilled H_2_O on UV irradiation. The time dependent decreasing emission indicates the degradation of the drug.

### 2.8 Planktonic antibacterial activity

The bacteria gram-positive *Enterococcus faecalis* (ATCC 29212) was used in the present study which generally can sustain the absence of oxygen, severe alkalinity and causes endodontic breakdown. The antibacterial activity test of Ca_2_Fe_2_O_5_ nanoparticles against *Enterococcus faecalis* were tested by Agar well-diffusion method.

Evaluation of minimum inhibitory concentrations (MIC) which is the least concentration of Ca_2_Fe_2_O_5_ nanoparticles required to suppress 99.0% of the growth of *Enterococcus faecalis.*

Wells of 6 mm diameter were punched on specific agar media with about 100 μl of pre-cultured test bacteria spread onto the agar plates and various concentrations of the Ca_2_Fe_2_O_5_ nanoparticles were loaded into the wells. Bacterial plates were incubated at 37 °C for 24 h, and Zone of inhibition were measured.

Further, six more agar plates with sub culturing of the wells were developed which were also incubated for 24 h at 37 °C. At the end of incubation duration, the growth of bacteria was observed by naked eye and Colony forming units (CFUs) was calculated on the grid. The Minimum Bactericidal Concentration **(**MBC) value is concluded as the lowest concentration that showed no apparent growth of *Enterococcus faecalis* on agar subculture.

### 2.9 Biofilm antibacterial activity

Each well of the sterile microtiter plate were filled with a mixture of 180 μL of BHI broth and overnight grown culture of *Enterococcus faecalis*. To each well Ca_2_Fe_2_O_5_ nanoparticles in differing concentrations were added so that final concentration of nanoparticle varied between 1 and 640 μg/mL. This was followed by incubation of microtiter plates for 24 h at 37 °C. The contents were gently washed with 3 mL of phosphate buffer saline (PBS) of pH 7.2 which ensures the removal of free planktonic bacteria. The adhering bacterial biofilm was then stained using crystal violet and the wells were filled with 95% (v/v) ethanol. Absorbance was quantified by UV–visible spectrophotometer at 530 nm.

The percentage of biofilm inhibition was calculated using the equation.

% Biofilm inhibition = [1- (OD at 530 of treated cells)/(OD at 530 of non-treated cells)] ×100.

The washed PBS samples serve as positive control and the absorbance was analyzed in UV spectrophotometer at 530 nm.

### 2.10 Complementary cryogenic SEM & EDAX studies

The study of surface morphology of Ca_2_Fe_2_O_5_ nanoparticles treated and untreated with *Enterococcus faecalis* using SEM technique provides strong evidence for the antimicrobial property. The biofilm of formed by *Enterococcus faecalis* was washed with 3 mL of 3% glutaraldehyde and 3 mL of phosphate buffer solution followed by drying with ethanol for 10 min. The prepared bacteria were mounted for imaging process and the surface morphology was investigated. The tested *Enterococcus faecalis* was also subjected to EDX spectrum analysis.

### 2.11 Cytotoxicity study on Allium Cepa cells

A set of five normal onion bulbs weighing around 40 g were grown at room temperature with a regular water renewal at 24 h. The emerging roots were approximately 3 cm long which were dipped in 0.01, 0.1, 1, 10 and 100 μg/mL Ca_2_Fe_2_O_5_ nanoparticles for 4 h. The roots were removed, washed with distilled water, hydrolysed for half an hour by 1 N HCl and stained for 10 min by dipping in Acetocarmine followed by incubation. 1 mm of the root was sliced and squashed over a slide under 100× magnification microscopic observation.

### 2.12 Cytotoxicity study on HeLa cell line using MTT method

**Scheme 1:**
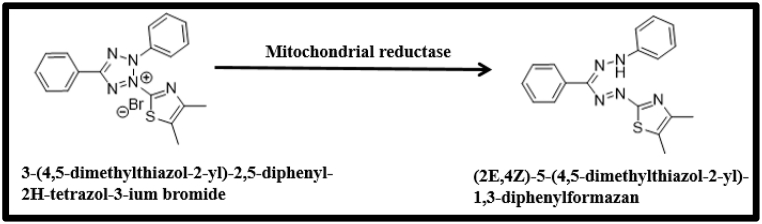
Structural representation of cleavage of the Tetrazolium ring by mitochondrial dehydrogenase enzymes.

IC_50_ value was determined using MTT method which incorporates bio indicator (3- [4, 5-dimethylthiazol-2-yl]-2, 5-diphenyl tetrazolium bromide) a yellow solid converted to a purple formazan by cleavage of the tetrazolium ring by mitochondrial dehydrogenase enzymes (Scheme 1) of viable cells. The intensity of the colour is spectrophotometrically monitored which an indicator of the degree of effect is caused by the test material.

## 3 Results and discussion

### 3.1 Structure characterization

#### 3.1.1 X-ray diffraction (XRD) analysis

**Fig. 2:**
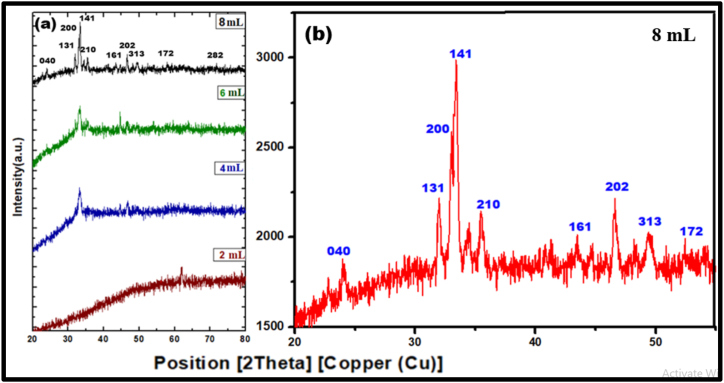
(a) PXRD pattern; (b) Enlarged view of PXRD with 8 mL of plant extract.

Fig. 2(a) depicts the XRD pattern of the Ca_2_Fe_2_O_5_ nanoparticles synthesized by eco-friendly green combustion route with the aid of *Acmella oleracea* plant extract in different volumes ranging from 2 mL to 8 mL and Fig. 2(b) an enlarged view of PXRD with 8 mL of plant extract.

A set of sharp peaks indicates the particles are in the Nano regime with good purity, reduced size, and crystalline nature. The Bragg's reflection 2θ peaks at around 22.75 ᵒ, 23.75ᵒ, 31.98 ᵒ, 33.39 ᵒ, 33.42ᵒ, 34.21ᵒ, 35.98ᵒ, 43.51ᵒ, 46.60ᵒ, 49.28ᵒ related to diffraction planes of (101), (111), (131), (200), (141), (210), (161), (202), (080), (172) respectively closely matches with the phase pure Brown-millerite structure as reported by Ref. [36] whose indexing is based on orthorhombic unit cell corresponding with Ref: JCPDS file no 00-047-1744. The crystallite size resulting from employing Scherrer's formula on the basis of (141) plane is determined as 13.8 and all other relevant parameters are listed in Table 1.

**Table 1:** Comparison of structural parameters of synthesized Ca_2_Fe_2_O_5_ Nano particles at four different concentrations of plant extract.

*hkl*	Volume of plant extract	D = 0.9λ/βcosθ (nm)	d = λx10^−10^/2sinθ (m)	δ = 1 × 10^−3^/D^2^	SF = 2π^2^/45 (3tanθ)^1/2^
141	2 mL	13.8	6.85	12.5	0.62
	4 mL	13.5	7.11	12.3	0.41
	6 mL	13.4	7.28	11.9	0.36
	8 mL	13.1	7.42	11.6	0.30

No other peaks corresponding to impurities were noticed. Variation of the volume of the plant extract increases the sharpness and intensity of the peaks indicating an enhanced crystallinity with no change in the reflection pattern which out rules any phase transformations. A list of various procedures adopted by different researchers along with physical features are listed in Table 2. It helps us to comparatively understand the various synthetic routes affecting the structural properties from previously published research articles.

**Table 2:** A summary of synthetic methodologies adopted to obtain Ca_2_Fe_2_O_5_ by different researchers.

SI no	Methodology	Calcination temperature	Crystallite size (nm)	Morphology	Eg (ev)	Ref
1	Sol-Gel	600	24.69	Agglomerated Flakes	2.0–2.2	[37]
2	Solution mixture method	750	14.47	Flaky structure	2.0	[38]
3	Solid state method	600	14.01	Spherical	3.05	[39]
4	Sol-gel auto combustion method	700	116	Crystalline	2.2	[40]
5	Chemical method	700	–	Crystalline	1.85	[41]
6	Sol-Gel method	500	–	Irregular beads	2.0	[40]
7	Combustion	600	13.1	Spherical	2.1	**Current work**

#### 3.1.2 Scanning electron microscopy (SEM) analysis

**Fig. 3:**
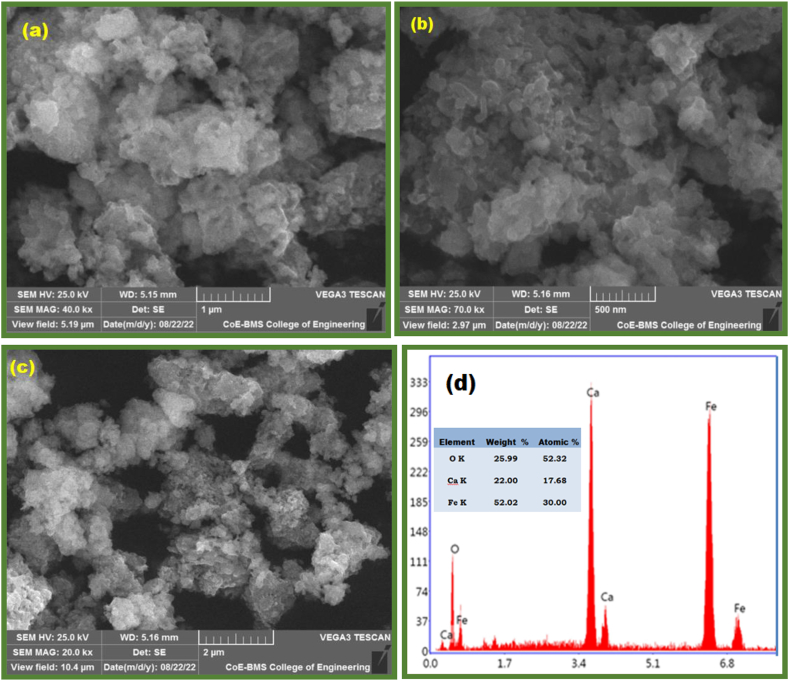
SEM image of Nano Ca_2_Fe_2_O_5_ (a–c) at different magnification and (d) EDAX image of Ca_2_Fe_2_O_5_.

Scanning electron microscopy image in Fig. 3(a–d) provides knowledge on the surface morphology of the near to spherically shaped particles. The image suggests an even distribution of micro structures near to spherical shape. A slight aggregation among the Nano structures of Ca_2_Fe_2_O_5_ is due to the large surface energy and magnetic interaction exerted during the direct contact of the particles [41].

##### 3.1.2.1 Fourier transform infrared (FTIR) spectra analysis

**Fig. 4:**
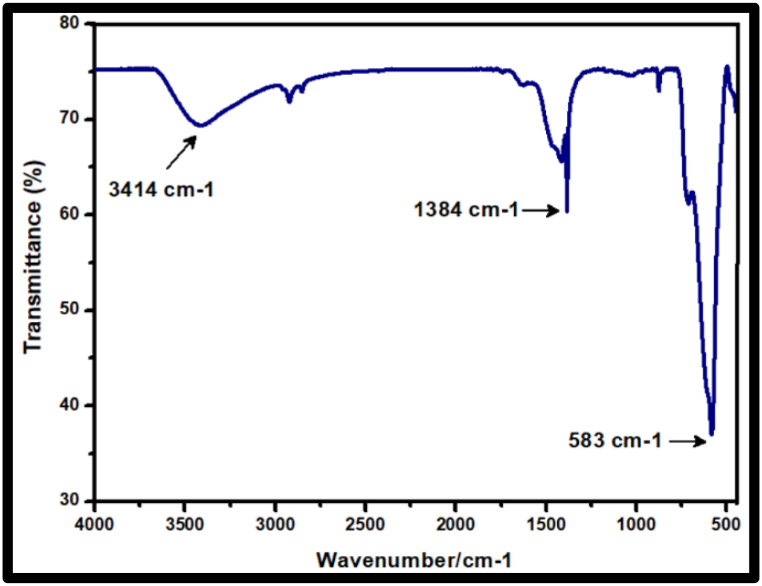
FTIR spectra of Nano Ca_2_Fe_2_O_5_.

Ca_2_Fe_2_O_5_ synthesized using *Acmella oleracea* plant extract shows intense FTIR peaks represented in Fig. 4. The peak around 583 cm^−1^ is attributed to Fe–O stretch [38] while 1384 cm^−1^ is caused due to Ca–O [42] and the signal at 3414 cm^−1^ is due to the surface hydroxyl groups in water adsorbed [43] on the surface.

### 3.2 UV–vis analysis of nano Ca_2_Fe_2_O_5_

A firm correlation is established linking optical properties and the electronic transitions [44] through UV absorbance spectroscopy.

**Fig. 5:**
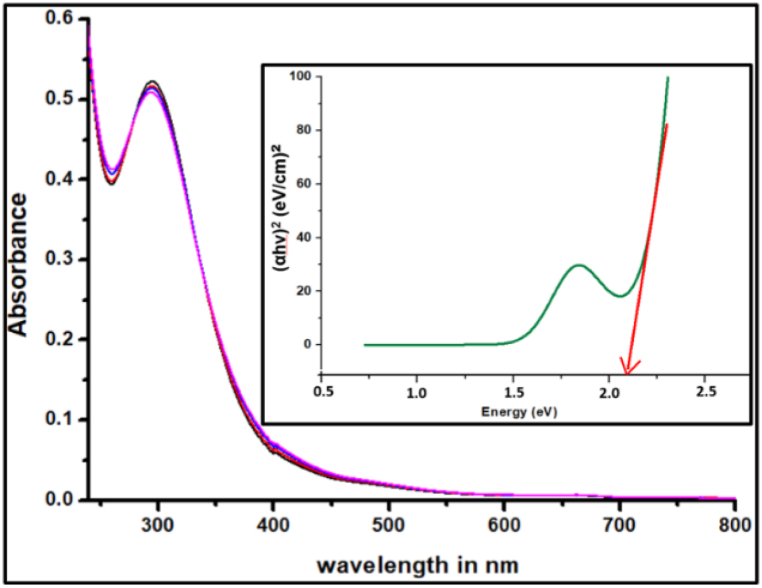
UV–visible absorption spectra (Inset Tauc's plot) for Ca_2_Fe_2_O_5_ nanoparticles.

A single characteristic absorption signal with a peak at about 295 nm and a declination from 349 nm to 430 nm wavelength as shown in Fig. 5 was obtained**.** The absence of any other peaks in the spectrum indicates an excellent purity of the obtained product. As no significant difference was found in the UV absorption peaks across all the samples of Ca_2_Fe_2_O_5_ prepared by varying the volume of the plant extract, a common Tauc plot serves to determine the direct band gap Eg.

Tauc plot based on UV–Visible spectra with (αhν)^2^ vs hν shown in Fig. 5 with the extrapolation of best linear curve to zero indicates a band gap of 2.1 eV, very close to that formerly reported for calcium ferrites [45].

### 3.3 Photocatalytic degradation of pharmaceutical pollutants

Owing to the capacity of absorbing UV radiations, photocatalytic activity of Ca_2_Fe_2_O_5_ was explored in degrading Clopidogrel bisulphate and Asprin as model pharmaceutical pollutants. Irradiation on solution of Clopidogrel bisulphate without any photo catalyst showed no notable change after 1 h. However, the addition of nano Ca_2_Fe_2_O_5_ catalyst causes a decrease in the intensity of absorption both the drugs which indicates an active degradation under applied conditions. With the onset of irradiation, the degradation of Clopidogrel bisulphate was evaluated at room temperature by monitoring an intense absorption between 200 and 260 nm centred at 220 nm.

**Fig. 6:**
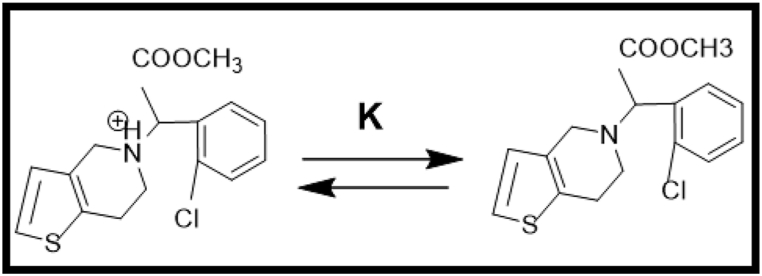
Equilibrium representation of protonated and deprotonated forms of Clopidogrel bisulphate.

The variation in pH can influence the chemical structure of the drug along with the surface ionisation properties of the catalyst and thereby the catalytic efficiency. A detailed study by Hugo Serra et al. [46] highlights the importance of pH dependent protonation and deprotonation equilibrium of Clopidogrel through the following representation in Fig. 6. Hence, the current study was examined at reasonably low pH 4, natural pH 7 and basic pH 10.

Similarly, photocatalytic activity of Nano Ca_2_Fe_2_O_5_ catalyst was tested for degrading Asprin through monitoring the decreasing intensities of two characteristic peaks at 224 nm and 296 nm with respect to the irradiation time. A decrease in the concentration of drug reflected in the depleting UV absorption intensity of the characteristic peak was noted.

**Fig. 7:**
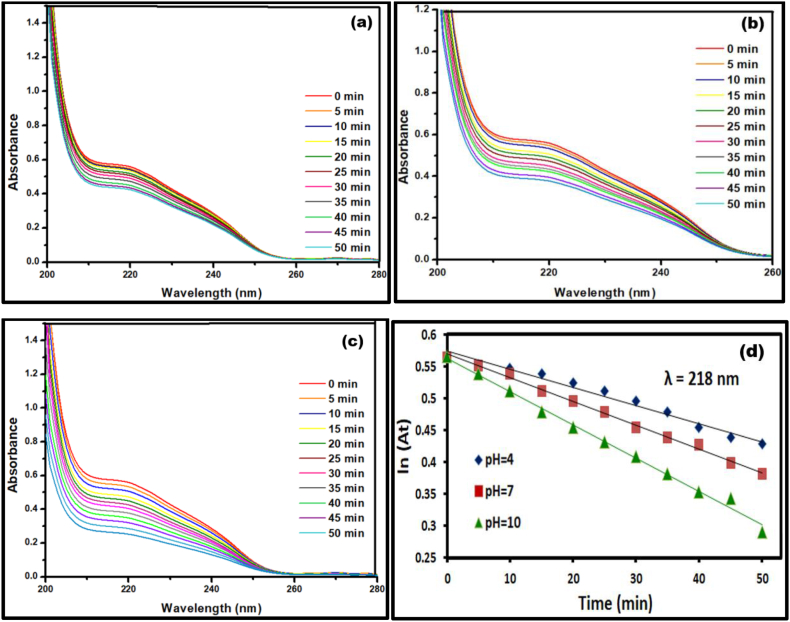
Photocatalytic degradation activity at (a) pH = 4 (b) pH = 7 (c) pH = 10 (d) corresponding first order kinetic fit of Clopidogrel Bisulphate at λ = 218 nm.

**Fig. 8:**
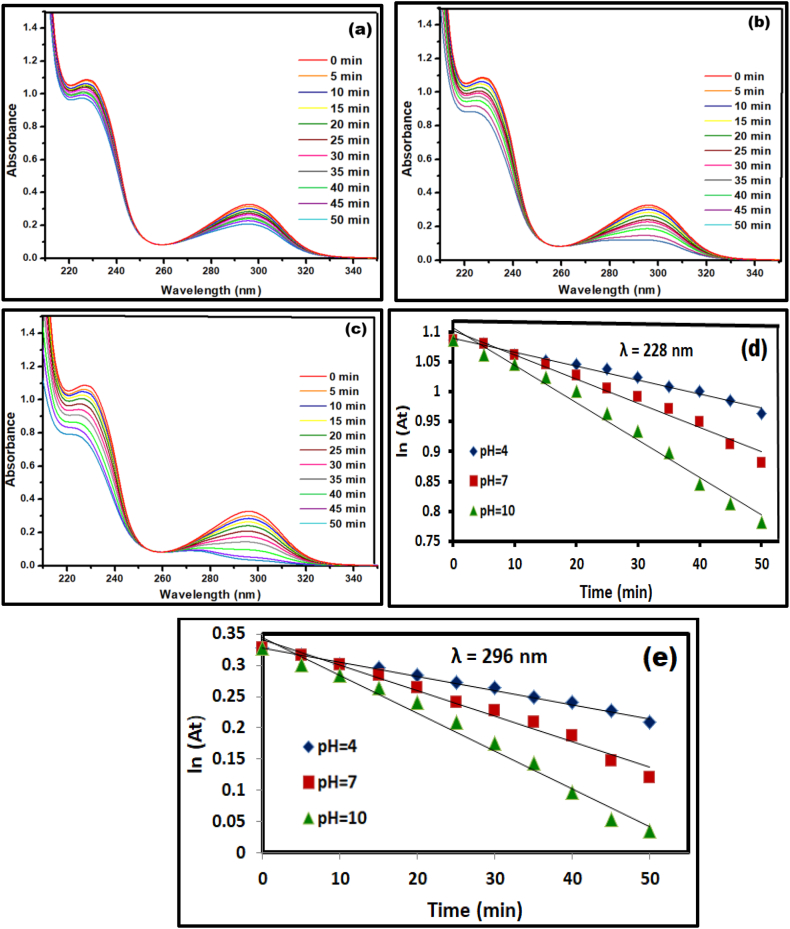
Photocatalytic degradation activity at (a) pH = 4 (b) pH = 7 (c) pH = 10.

In each of these cases as depicted in Fig. 7, Fig. 8, degradation follows a pseudo-first order rate law calculated using ln (A/A_0_) k = −t, and degradation efficiency by employing Lambert Beer's Law, E% = 100 (A_0_–A/A_0_) [47] with k, A_0_ and A representing the rate constant, absorbance at time t = 0 and absorbance at different time intervals respectively. The first-order rate constants are calculated using slope k from the best-fit line of the data points with an average regression coefficient of 0.995 which is close to unity indicating a good linearity of the model and is tabulated in Table 3.

**Table 3:** Rate constants, Regression co-efficients and percentage degradation efficiencies for Clopidogrel bisulphate and Asprin at varying pH.

Drug	pH	Rate constant x 10^−3^ (min^−1^)		R^2^	% Degradation efficiency
		λ = 218			
Clopidogrel Bisulphate	4	−0.0030		0.9902	81.66
	7	−0.0038		0.9985	83.94
	10	−0.0052		0.9979	89.85
Asprin	pH	λ = 224	λ = 296		
	4	−0.0031	−0.0028	0.9892	78.04
	7	−0.0042	−0.0035	0.9913	81.32
	10	−0.0058	−0.0040	0.9988	88.25

It is observed that subtle changes in the UV absorption due to degradation are noted at pH of 4, while a better degradation at pH of 7 and maximum activity at pH 10. At a low pH, a high concentration of H^+^ ions inhibit the generation of hydroxyl anions and thereby the hydroxyl radicals which drives the photocatalytic activity. The current study showing enhanced photodegradation rate in the alkaline medium is similar to the scenario reported by Maged El-Kemary et al. [48] and the photoactivity reported earlier by Ca_2_Fe_2_O_5_ by organic effluents [49] which follows a typical photocatalysis route of absorbing photons to generate a hole in the valence band and an extra electron in the conduction band leading to reactive oxygen species capable of attacking the target pharmaceutical pollutant and dissociating it in to CO_2_ and H_2_O [50].

In the course of degradation, production of ROS was confirmed by scavengers [51] and species trapping experiments [49] indicated previously for calcium ferrate. The attack of electron or hole on the drug molecule adsorbed by the surface of nanoparticle causing an indirect oxidation has always been recognised as the first major step in photo degradation [52,53]. In the indirect oxidation process, the superoxide radicals generated by the photo excited catalyst combines with proton in the water and releases hydroxyl radicals [54] which is in accordance with enhancement of degradation rate in basic media depicted in Fig. 7, Fig. 8.

Corresponcf Asprin (d) λ = 224 nm (e) λ = 296 nm.

(1) $${Ca}_{2}{Fe}_{2}O_{5}\overset{UVradiation}{\to }{{Ca}_{2}{Fe}_{2}O_{5}}^{*}\;\left(energy\right)$$

(2) $${{Ca}_{2}{Fe}_{2}O_{5}}^{*}\;\left(energy\right)\to {Ca}_{2}{Fe}_{2}O_{5}\left(h^{+}+e^{-}\right)$$

(3) $${Ca}_{2}{Fe}_{2}O_{5}\left(e^{-}\right)+O_{2}\to {O_{2}}^{∙-}\left(super\;oxide\;radical\right)$$

(4) $${O_{2}}^{∙-}+H_{2}O\to {OH}^{-}+OH$$

Accordingly, the catalytic activity of Ca_2_Fe_2_O_5_ in the present study can be represented in the equations (1), (2), (3), (4) that follow.

### 3.4 GC–MS results

**Fig. 9:**
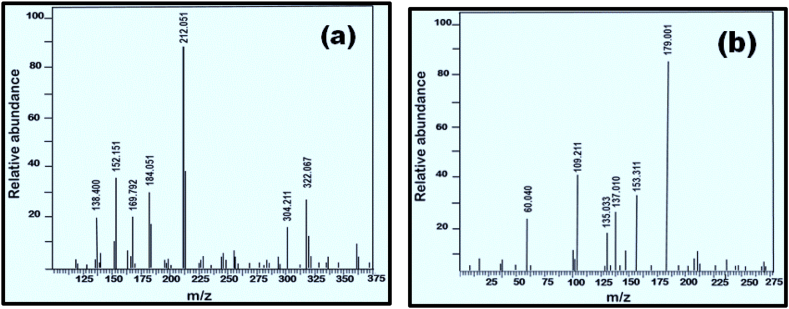
Liquid chromatography-mass spectrometry (LC-MS) spectra for degradation products of (a) Clopidogrel Bisulphate (b) Asprin.

Products of degradation of Clopidogrel bisulphate and Asprin were identified through fragments obtained by GC–MS peaks shown in Fig. 9(a) and (b).

**Fig. 10:**
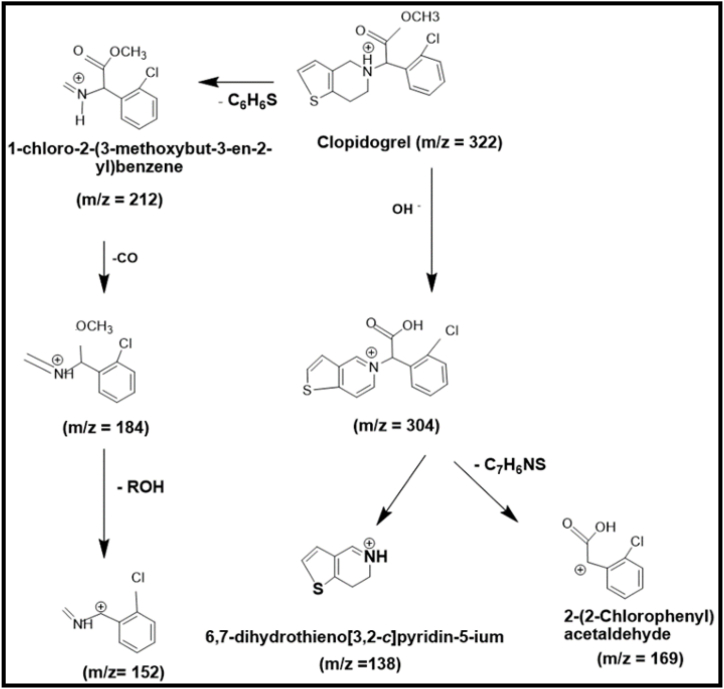
A plausible pathway for Ca_2_Fe_2_O_5_ catalysed photocatalytic degradation of Clopidogrel bisulphate.

Fig. 9(a) shows the molecular ion peak [M+H] + of Clopidogrel bisulphate indicated by the peak at *m*/*z* of 322 and all other peaks at *m*/*z* of 212, 322, 184, 152, 304, 138 and 169 which closely resembles the observations by Dhara et al. [55]. However, tracing of effect of the mass fragmentation on Chlorine containing part of the drug through isotopic patterning is out of the scope of the present study. Based on the fragmentation pattern reported by Dhara et al. [55] a plausible mechanism for the degradation for the current study is shown in Fig. 10.

**Fig. 11:**
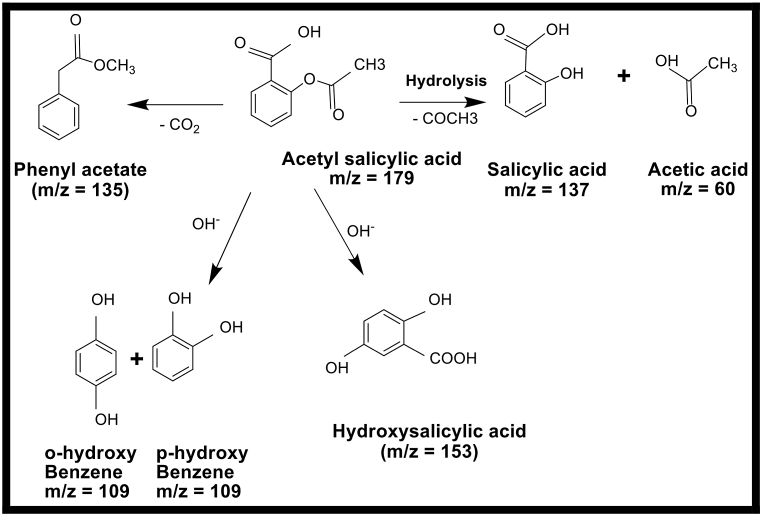
A plausible pathway for Ca_2_Fe_2_O_5_ catalysed photocatalytic degradation of Asprin.

Fig. 9(b) shows the molecular ion peak [M+H] + of Asprin indicated by the peak at *m*/*z* of 179 with all other peaks closely resembling to those reported by Lezhuo Li et al. [22,23] which includes *m*/*z* 60, 135,137, 109, 153 and 179 caused due to intermediates that eventually decomposes to carbon dioxide and water. The products and the degradation pathway for Asprin is outlined in Fig. 11 based on GC-MS studies and previous studies reported [23].

### 3.5 Fluorescence analysis

**Fig. 12:**
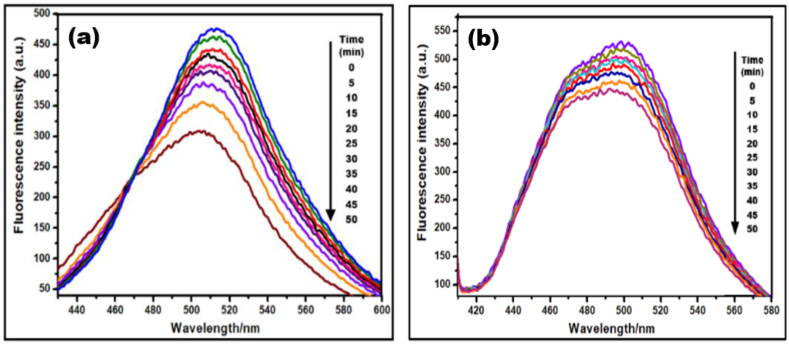
Fluorescence emission spectra Quenching effects of (a) CLP drug (10 mg/L) (b) ASP drug (10 mg/L) in the presence of Ca_2_Fe_2_O_5_ nanoparticles (20 mg/L).

In view of higher degradation response at pH 10, fluorescence emission spectra of Clopidogrel bisulphate and Asprin was recorded with excitation wavelength 440 nm in the presence of Ca_2_Fe_2_O_5_ nano particles to explore the degradation behaviour. The emission peak between 400 and 600 nm in Fig. 12 (a-b) clearly shows the emission intensity of both the drugs decreasing on UV irradiation as a function of degrading duration. However, a slight peak position shifting in case of Clopidogrel bisulphate towards shorter wavelength from 518 to 502 nm may be attributed to reshuffled electron density and an increased dipole moment during electronic excitation that disturbs the equilibrium of solvent molecules. This is followed by excited-state solvent relaxation that decreases the energy level of the excited state causing a lower energy emission [56].

### 3.6 Planktonic antibacterial activity

*Enterococcus faecalis* is one of the prominent bacteria posing endodontic therapeutic difficulties with high resistance to commonly available antibiotics. It is extensively found to block root canals causing apical periodontitis [57] and can survive under harsh conditions.

**Fig. 13:**
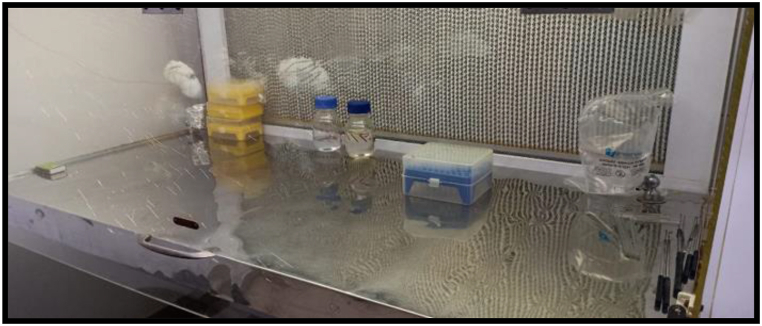
*Enterococcus faecalis* preserved in laminar air flow chamber.

Calcium based Nano particles are studied for bactericidal properties across various species over decades. Recent reports by *Sanaa. G* et al. [58] showed excellent results towards bacterial inactivation. In view of many successful endodontic irrigants such as antiseptics [59] and new generation of nano-antimicrobial agents [60] becoming popular against *Enterococcus faecalis*, antibacterial activity of Ca_2_Fe_2_O_5_ was evaluated in the current study which was preserved in laminar air flow chamber shown in Fig. 13.

**Fig. 14:**
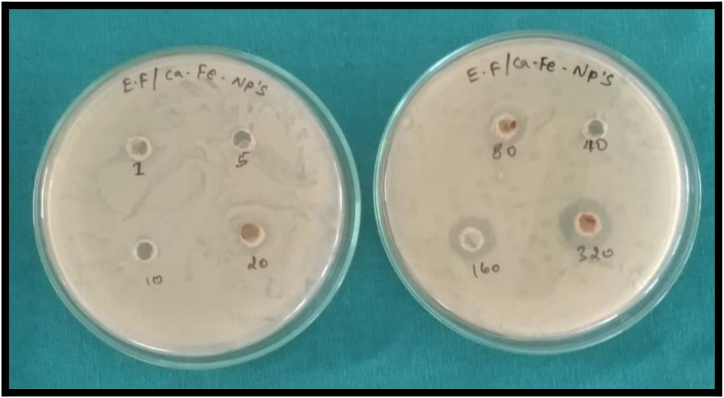
Inhibition Zone of Ca_2_Fe_2_O_5_ nanoparticles with concentrations (1) 5 μg/mL, (2) 5 μg/mL, (3) 10 μg/mL (4) 20 μg/mL (5) 40 (6) 80 (7) 160 (8) 380 and (x). Cipro floxacin as positive control against *Enterococcus faecalis*.

Ca_2_Fe_2_O_5_ nanoparticles were evaluated for bacterial inhibitory behaviour towards an overnight culture of *Enterococcus faecalis* at 37 °C under aerobic conditions by employing Agar well-diffusion method shown in Fig. 14 using 0.5 MC of BHI Agar media.

It is apparent that Ca_2_Fe_2_O_5_ nanoparticles moving from a concentration of 1 μg/mL to 80, 160 and 320 μg/mL has inhibited the growth of *Enterococcus faecalis* indicated by the zone of inhibition values 10 mm ± 0.5, 12 mm ± 0.5 and 14 mm ± 0.5 respectively.

A comparative analysis of bactericidal action of various nano materials against *Enterococcus faecalis* is presented in Table 4.

**Table 4:** Summary of Nano materials active against *Enterococcus faecali*s in literature.

SI No	Nanomaterial	Size of nanomaterial (nm)	Zone of inhibition (mm)	Ref
1	E. Blanda-chitosan-CuO	30.76	18.00	[61]
2	Curcumin capped ZnO	20 ± 5	20.30	[62]
3	Bi2O3-CNT	44.83	21.00	[63]
4	Selenium nanoparticles	30–50	28.50	[64]
5	TiO2 nanoparticles	10–49	21.00	[65]
6	Ca2Fe2O4	13.1	14.00	Current work

**Fig. 15:**
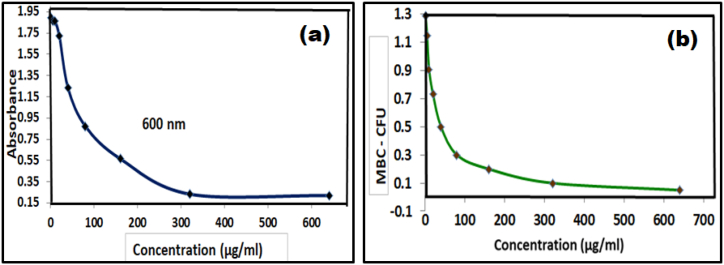
Graphical representation of (a) MIC and (b) MBC against *Enterococcus faecalis*

The anti-bacterial action of Ca_2_Fe_2_O_5_ nanoparticles against *Enterococcus faecalis* was further reinforced by the evaluation of Minimum inhibitory concentrations (MIC) determined by micro dilution assay and Minimum Bactericidal Concentration (MBC) as indicated by Fig. 15(a–b).

*Enterococcus faecalis* is evaluated previously for both the above stated assays using Silver nano particles [66] and Calcium Peroxide [67]. Also, Ca_2_Fe_2_O_5_ porous powder has been used in detecting various phases of fungal proliferation of Candida utilis [68] to explore the cell susceptibility. However, MIC and MBC of Ca_2_Fe_2_O_5_ nanoparticles against *Enterococcus faecalis* is investigated for the first time ever.

The microplates were maintained under same conditions as stated for zone inhibition tests. A series of dilutions of bacteria were prepared to determine the lowest concentration which exhibits antibacterial properties. A spectrophotometric monitoring of optical density at 600 nm indicates a repressed growth of the bacteria with an average value of MIC being 0.103 calculated by the data listed in Table 5.

**Table 5:** MIC calculated by decreasing absorbance indicating suppression of bacterial growth.

Concentration (μg/ml)	1	5	10	20	40	80	160	320	640
**MIC (OD)**	1.89	1.86	1.86	1.72	1.23	0.87	0.56	0.23	0.22

The investigation was further extended to determine the Minimum Bactericidal Concentration (MBC) in terms of colony forming units (CFUs) on the grids, using a sub culture of the above said bacteria grown on sterile agar plates that will display no perceivable growth of the colony that was confirmed through the data listed in Table 6.

**Table 6:** MBC calculated by suppressed Bacterial growth at different concentrations of *Enterococcus faecalis*.

Concentration (μg/ml)	1	5	10	20	40	80	160	320	640
**MBC - CFU**	1.29	1.15	0.91	0.73	0.5	0.3	0.2	0.1	0.05

### 3.7 Biofilm antibacterial activity

**Fig. 16:**
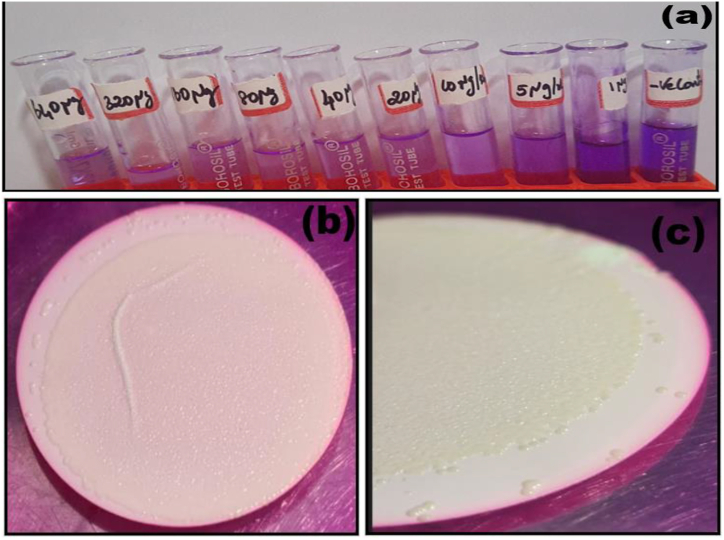
Antibiofilm activity of the prepared Ca_2_Fe_2_O_5_ (a) producing blue coloured suspension (b) and (c) *Enterococcus faecalis* biofilm visualised under microscope at different magnifications. (For interpretation of the references to colour in this figure legend, the reader is referred to the Web version of this article.)

Bacterial biofilm is an adherent structured agglomeration that can resist destructive influences more effectively. The efficacy of traditional antibiotics is therefore lesser on biofilms compared to planktonic bacteria [69]. Pioneering results incorporating modified Ag nanoparticles [70] has provided successful solutions towards dental biofilms. However, in view of the limitations of these reagents and an urgent requirement to develop more number of antibiofilm nano agents, Ca_2_Fe_2_O_5_ nanoparticles was tested for antibiofilm effectiveness by adopting crystal violet staining test tube method [71] and the repression was monitored using UV spectrophotometer owing to its simplicity and accuracy. *Enterococcus faecalis* inoculation in the absence of Ca_2_Fe_2_O_5_ nanoparticles caused a film across the air–liquid junction that was adherent towards the walls of the test tube. The film emerged as a blue coloured ring on crystal violet staining and gradually dissolved to form a blue coloured suspension on adding absolute ethanol as displayed in Fig. 16(a–c) and Table 7.

**Table 7:** Absorbance monitored at 530 nm during crystal violet assay Crystal Violet staining assay.

Concentration (μg/ml)	1	5	10	20	40	80	160	320	640	Control
**OD at 530 nm**	0.187	0.172	0.168	0.162	0.162	0.119	0.087	0.063	0.069	0.820

**Fig. 17:**
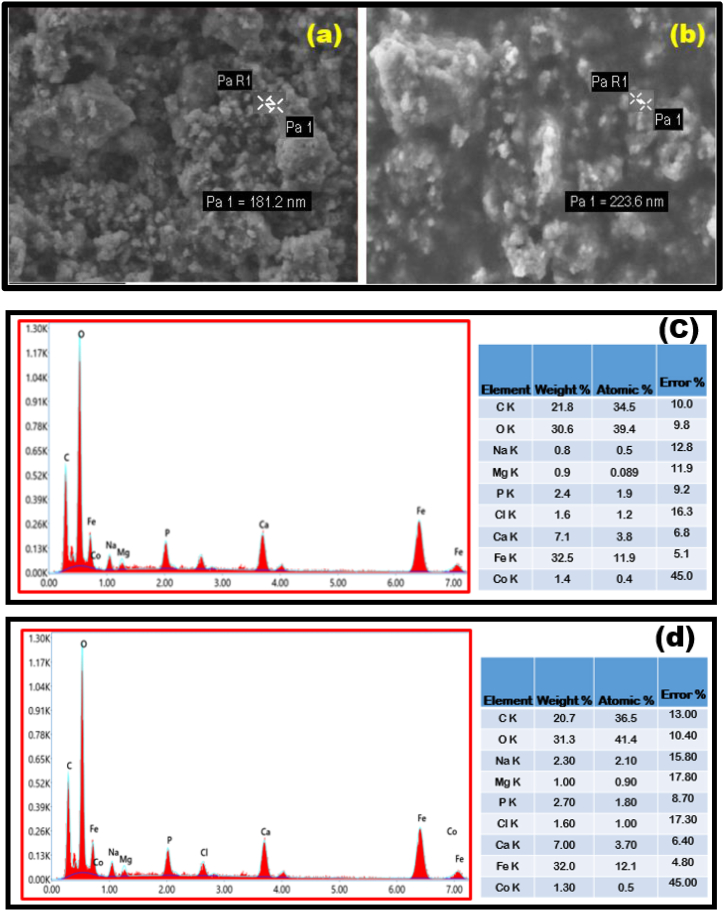
Cryogenic-SEM of (a) Ca_2_Fe_2_O_5_ nanoparticles -control (b) *Enterococcus faecalis* bacteria deposited on Ca_2_Fe_2_O_5_ nanoparticles (c) EDAX spectra of Ca_2_Fe_2_O_5_ nanoparticles (d) EDAX spectra of Ca_2_Fe_2_O_5_ nanoparticles treated with *Enterococcus faecalis*bacteria

The current study is the first report to explore the antibiofilm properties of Ca_2_Fe_2_O_5_ nanoparticles against *Enterococcus faecalis* and an insight into the mechanistic action can be enhanced through Cryogenic-SEM and EDX studies. No single explanation for the mechanism of nanoparticle aided antibacterial activity has provided an unparalleled theory. Metal nanoparticles involved in the degrading the enzymes, cellular proteins, genetic coding along with production of reactive oxygen species (ROS) [72] and invasion into bacterial cells through transformation of phospholipids in the cell wall thereby changing the surface charge [73] are the most convincing reports. Surface characteristics and the morphological changes due to interaction of Nano particles with the bacteria can be determined by Cryogenic-SEM and EDX. In the current scenario, Nano Ca_2_Fe_2_O_5_ and bacteria adhered to it is shown in Fig. 17(a and b) where an increase in the size of the Nano particles from 181 nm to 223.6 nm is apparent. The expansion can be attributed to internalization of bacteria or agglomeration of Nano particles around the bacteria thereby making them unavailable to form colonies and hence a reduction in biofilm. The corresponding EDX shown in Fig. 17(c and d) of Nano Ca_2_Fe_2_O_5_ before and after treating with bacteria reflects a small variation of elements due to the association of nanoparticles with bacteria.

### 3.8 Toxicity studies of Ca_2_Fe_2_O_5_ nanoparticles

#### 3.8.1 Cytotoxicity study on Allium Cepa cells

Toxicity of nano materials has always been a concern and controversial research area. Biological assessment such as cytotoxicity through Allium Cepa root cell test [74] can serve as markers to ensure biocompatibility.

**Fig. 18:**
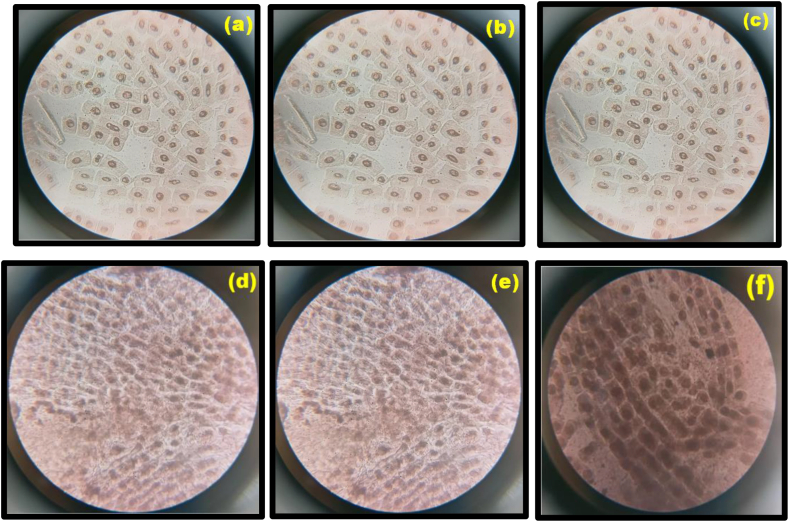
Microscopic images of Allium Cepa cells (a) untreated and treated with (b) 0.01 (c) 0.1 (d) 1, (e) 10 and (f) 100 μg/mL concentrations.

Pioneering results are reported systematically by Priyanka et al. [75] on the impact of nanoparticles employing Allium Cepa root cell test whose cytotoxic evaluation was based on the microscopic view of root tip cells. Cytological changes displayed in Fig. 18(a–f) are witnessed in the root cells after interacting for 5 h with Ca_2_Fe_2_O_5_ nano particles at concentrations ranging from 0.01, 0.1, 1, 10, and 100 μg/mL. The results clearly indicate that Ca_2_Fe_2_O_5_ nano particles are safer till 10 μg/mL after which its biocompatibility is doubtful. However, the dose dependent influence on cell morphology and chromosomal aberrations can provide information on disruption of cells which is under progress in our lab.

#### 3.8.2 Cytotoxicity study on HeLa cell line

**Fig. 19:**
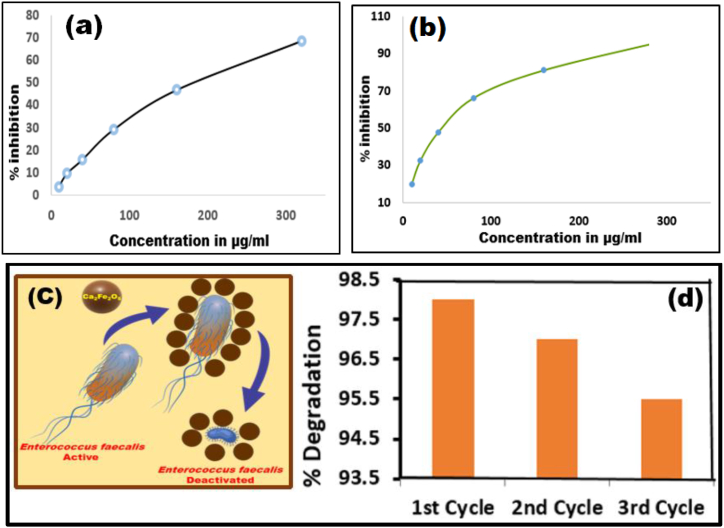
Sigmoid dose-response curve for (a) Ca_2_Fe_2_O_5_, (b) Standard Doxorubicin (c) Representation of Ca_2_Fe_2_O_5_ nanoparticles induced bacterial toxicity (d) extent of reusability of Nano Ca_2_Fe_2_O_5_ for three successive cycles.

The cytotoxicity of Ca_2_Fe_2_O_5_ nano particles is assayed utilising HeLa cell line procured from cervical cancer cells employing MTT method [76] known for its sensitivity, simplicity, rapidity and repeatability. Table 8 displays nonlinear regression data leading to IC_50_ through sigmoidal curve representing dose-response curve. A moderately high IC_50_ value of 170.94 μg/mL in Fig. 19 (a) when compared with Doxorubicin with 59.56 μg/mL in Fig. 19(b) indicates a moderate inhibiting behaviour on HeLa cell proliferation. However, the inhibitory effect can only be confirmed by Flow Cytometric studies which is under progress in our lab.

**Table 8:** Statistics for inhibitory potential of Ca_2_Fe_2_O_5_.

Compound name	Conc. μg/ml	OD at 590 nm	% Inhibition	IC50 μg/ml
Control	0	0.673	100	
Ca2Fe2O5	10	0.647	3.86	170.94
	20	0.608	9.658	
	40	0.566	15.89	
	80	0.476	29.27	
	160	0.358	46.80	
	320	0.211	68.64	
Doxorubicin				
Compound name	Conc. μg/ml	OD at 590 nm	% Inhibition	IC50 μg/ml
Control	0	0.661	100	59.56
	10	0.530	19.8	
	20	0.445	32.67	
	40	0.346	47.65	
	80	0.224	66.11	
	160	0.125	81.08	
	320	0.004	99.39

### 3.9 Antimicrobial effect of Ca_2_Fe_2_O_5_ nanoparticles

The exterior layer of the Gram-positive bacteria is encompassed by peptidoglycan extremely sturdy across the layers of which lengthy anionic polymers of teichoic acids are threaded [77]. The positive component of Ca_2_Fe_2_O_5_ nanoparticles**,** especially Iron due to the higher oxidation state of Fe [41] is attracted by an electrostatic force towards the bacterial cells and hence its morphology is altered. In addition, this process agrees well with the aggregation of Nano particles observed around the bacteria in Fig. 17. Therefore, the electrostatically bound Nano Ca_2_Fe_2_O_5_ to the bacterial cell wall hinders its activity as shown in **19 (c)** which can explain all the above stated biological activities. However, the mode of action at the cellular level can be only be confirmed by transmission electron microscopic studies of the cellular membranes which in progression in our lab.

## 4 Reusability of Ca_2_Fe_2_O_5_

Ferromagnetic Iron being one of the component of Ca_2_Fe_2_O_5_, was segregated with the aid of an external bar magnet and repeatedly cleaned with distilled water and ethanol followed by drying at 100 °C. Photocatalytic degradation experiment was repeated for three successive times and was found to be close to concurrence as 98%, 97% and 95% shown in Fig. 19(d) indicating the retention of activity.

## 5 Conclusions

*Acmella oleracea* plant extract as a fuel offers an efficient method to prepare Nano-sized Ca_2_Fe_2_O_5_ by combustion technique involving calcination at 600 °C. The prepared nano compound was characterised using XRD, SEM, UV, IR and implemented in the photocatalytic degradation of overused cardiovascular drugs which pose a serious to the environment as pollutants. The photocatalytic degradation efficiency was found to be over 80% with 10 mg/L photocatalyst. The prepared nano material was also tested for enhancement of bactericidal action against *Enterococcus faecalis*. The photocatalytic properties were observed to be pH dependent and antimicrobial property concentration dependent. Bacterial inhibitory behaviour was assessed by Minimum inhibitory concentrations (MIC) and Minimum Bactericidal Concentration (MBC) which was further confirmed by antibiofilm evaluation. Toxicity of Nano Ca_2_Fe_2_O_5_ was evaluated by *Allium Cep*a root cell test and HeLa cell line test indicating non-toxicity of the compound. As the current work involves innocuous nano compound, it extends new possibilities in developing eco-friendly pharmaceutical waste water treatment, anti-endodontic bacterial agents and would benefit further studies in this area.

## Author contribution statement

Neelam Patil Radhika: Conceived and designed the experiments. Malini S: Conceived and designed the experiments; Performed the experiments; Wrote the paper. Kalyan Raj, Shylaja K. R: Contributed reagents, materials, analysis tools or data. K.S. Anantharaju, Abhishek Appaji: Analyzed and interpreted the data.

## Funding statement

One of the authors, Malini.S would like to greatly acknowledge the financial support obtained under the Faculty research promotion scheme (FRPS), BMS College of engineering, Bangalore, India. (Project Grant R&D/FRPS/2022-23/CHY/04).

## Data availability statement

Data included in article/supplementary material/referenced in article.

## Declaration of competing interest

Authors have declared that no conflicts of interests exist.
